# An ossifying bridge – on the structural continuity between the Achilles tendon and the plantar fascia

**DOI:** 10.1038/s41598-020-71316-z

**Published:** 2020-09-03

**Authors:** Johann Zwirner, Ming Zhang, Benjamin Ondruschka, Keichi Akita, Niels Hammer

**Affiliations:** 1 Department of Anatomy, University of Otago, Dunedin, New Zealand; 2 Department of Clinical Anatomy, Tokyo Medical and Dental University, Tokyo, Japan; 3 Institute of Legal Medicine, University Medical Center Hamburg-Eppendorf, Hamburg, Germany; 4 Department of Macroscopic and Clinical Anatomy, Medical University of Graz, Graz, Austria; 5 Department of Orthopaedic and Trauma Surgery, University of Leipzig, Leipzig, Germany; 6 Fraunhofer IWU, Dresden, Germany

**Keywords:** Bone, Ligaments, Tendons, Biomedical engineering

## Abstract

Highly regular aligned trabeculae are found in the superficial posterior and inferior calcaneus appearing to connect the Achilles tendon (AT) to the plantar fascia (PF) in a bridge-like manner. This provides a morphological basis for the stretching-based heel pain treatment. However, the continuity of collagen fibres between the AT and the PF remains debated controversially to date. The given study morphologically investigated the AT-calcaneus-PF complex using histology and plastination. Moreover, the AT-calcaneus-PF complex was biomechanically mapped based on 13 sub-regions with a total of 76 tested samples. Regular calcaneal trabeculae were surrounded by tendon-like collagen fibre bundles and adipocytes. The orientation of calcaneal trabeculae was further closely related to the course of the PF collagen fibre bundles. The pooled biomechanical analysis revealed low elastic moduli (minimum = 4 MPa) and ultimate tensile strengths of the decalcified calcaneal samples (minimum = 0.4 MPa) and the calcaneal periostea (minimum = 2 MPa) and high respective values (elastic modulus maximum of 144 MPa; ultimate tensile strength maximum of 29 MPa) for the PF samples compared to the other sub-regions. This study provides structural evidence for a morphological connection between the AT and PF via the highly aligned calcaneal trabeculae of the posterior calcaneus. The AT-calcaneus-PF complex was biomechanically mapped to allow for an assessment of its site-dependent mechanical characteristics.

## Introduction

Plantar fasciitis is the most frequent cause of inferior heel pain and affects approximately one percent of the adult population^1^. A variety of stretching techniques including both the plantar fascia (PF) and Achilles tendon (AT) have been established as effective non-surgical treatment options to alleviate the plantar fasciitis pain patients describe as piercing, searing or throbbing^2^. Night splints, which keep the foot in a neutral or slightly dorsiflexed position while the patient rests are another stretching-related option, involving the concept of an AT-PF connection to mitigate the PF-related pain. Moreover, it was shown that high-load strength training including repetitive heel rises out of a dorsiflexed position positively influenced the therapy outcome of plantar fasciitis pain patients^3^. A shortened or tight AT, however, is an established risk factor for plantar fasciitis, once more underlining a close functional relationship of the two collagenous structures^4,5^. From a morphological perspective, highly regular aligned trabeculae were noted in the calcaneus that seemed to anatomically connect AT and PF in a bridge-like manner^6^. These published findings raised the question whether the AT and PF are two structurally different tissues, inserting separately into the calcaneus from two ends, or if the AT and PF resemble one continuing collagenous structure, which is secondarily divided by the calcaneal ossification during life^6^. Supporting the latter, it was shown that AT and PF are connected by thick collagen fibres in neonates, which quantitatively diminished during life and could not be detected in late(r) adulthood as they were likely integrated into the bony calcaneus^7^. Integration of the formerly continuous tendon into the posterior calcaneus, which then further subdivides into the distal PF and the proximal AT likely allows for a more efficient energy use during push-off movements of the foot and shows an adaption to bipedalism^8^. The calcaneal periosteum, which receives fibres from both AT and PF is only vaguely accepted as a continuous morphological connection between the two structures^7,9–11^, certainly with insignificant load-bearing characteristics. Also, it was noted that the calcaneal periosteum in the area of the highly regular trabeculae that possibly connect AT and PF differs from the remaining calcaneal periosteum, indicating the potential previous continuity of the two tendons^9^. If the AT and PF form one continuous structure which becomes separated throughout development, it can be hypothesized that the AT represents the proximal part, the regular calcaneal trabeculae including the calcaneal periosteum the intermediate part and the PF the distal part of one united tendon of the gastrocnemius and soleus muscle. To shed light on the structural connection between the AT and the PF via the calcaneus this study followed two aims: Firstly, the proximal–distal transition of the AT-calcaneus-PF complex should be morphologically investigated continuously between the formation of the AT starting at the distal calf and ending at the insertion of the PF at the heads of the metatarsal bones. Secondly, it aimed at establishing a biomechanical testing setup for this heterogenous complex, which allows for a uniform investigation of the structural organization by means of a materials testing approach to subsequently enable the structural analysis of the same from a materials testing perspective.

## Materials and methods

### Harvesting and sample preparation for mechanical testing

A total of nine complexes comprising of the human AT, adjacent calcaneus and PF (subsequently referred to as AT-calcaneus-PF complex) were retrieved post mortem (mean age 74, age range 28 to 93). One of the complexes was used for histological assessment (28-year-old male), two for plastination (87-year-old female, corresponding to one left and one right complex) and six for mechanical testing (four phenoxyethanol-based embalmed cadavers^12^, left and right complex was used of two cadavers, two right complexes of two other cadavers, mean age 82 years, 3 males, 1 female). The histologically processed AT-calcaneus-PF complex was retrieved at the Institute of Legal Medicine, University of Leipzig, Germany during leg preparation and the remaining ones were acquired from bequeathed cadavers for medical education and research purposes at the Department of Anatomy of the University of Otago in Dunedin, New Zealand. The University of Otago Ethics Committee approved this study (approval number H17/20), in conjunction with Maori consultation being sought from the Ngai Tāhu Research Consultation Committee and the Ethics committee of the University of Leipzig, Germany Study approved the tissue sampling (protocol number 486/16-ek).

### Dissection, decalcification and sample retrieval

**Figure 1:**
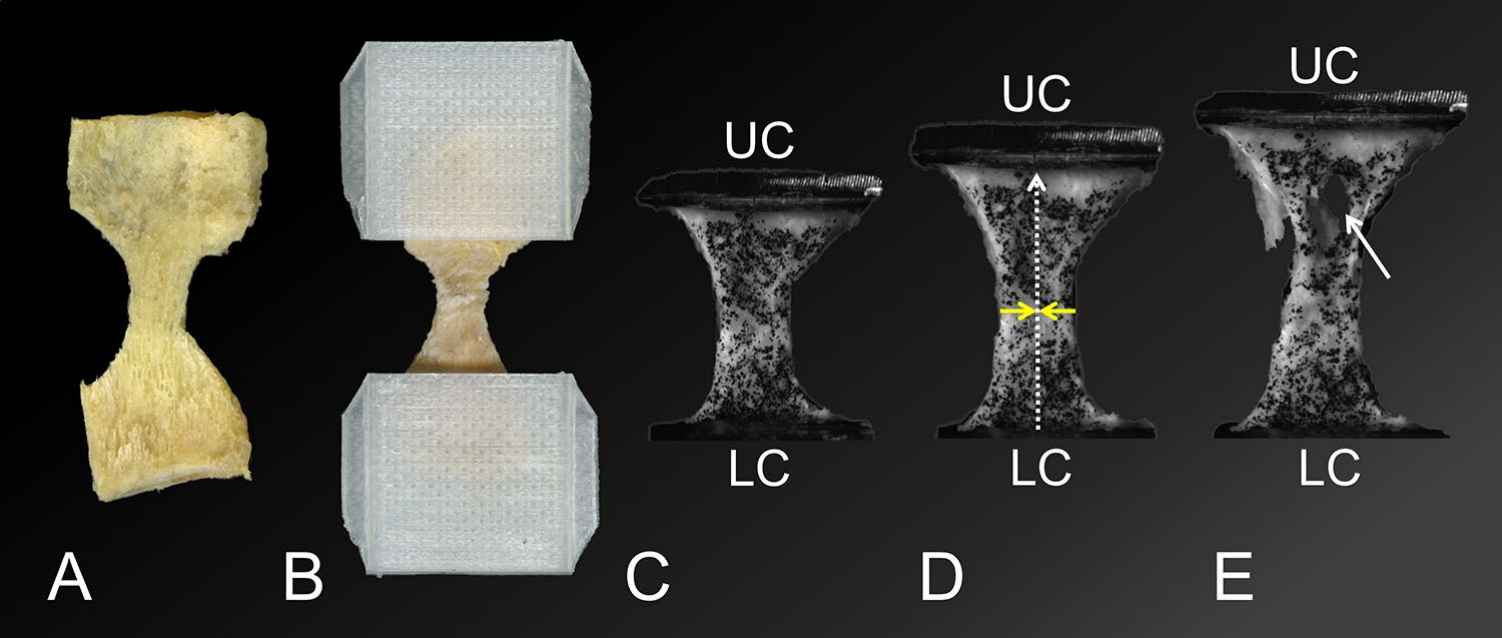
The images display the preparation steps of samples for tensile testing and the subsequent uniaxial load application at different testing stages. (**A**) A lateral calcaneal periosteum sample is depicted after it has been cut into a “dog bone” shape (view from inside). (**B**) The sample from A was mounted into 3D-printed squeezing clamps (view on the sample from outside). (**C**) Speckled and mounted sample in the material testing machine, upper (UC) and lower (LC) clamp. (**D**) A load is applied to the sample, indicated by the UC crosshead displacing cranially. The sample elongates (represented by the dotted arrow) and narrows in the ‘shaft’ region of the dog bone, indicated by the yellow arrows. (**E**) Continuous load application causes tearing of the sample (indicated by the white arrow).

Following the retrieval, the six AT-calcaneus-PF complexes of the mechanical testing group were prepared for decalcification as follows: First, the AT was exposed carefully until the calcaneus was reached distally, consecutively removing all surrounding tissues except for the periosteum. Second, the AT was proximally transsected, distally to the macroscopically last visible muscle fibres of the soleus muscle. Thereafter, the PF was exposed and mobilized from the heads of the metatarsal bones distally to the calcaneus proximally with a sharp scalpel. Following this, the lower half of the calcaneus was cut with an ultrasound knife (PIEZOSURGERY®, Mectron, Saline, MI, USA) with subsequent blunt removal of trabecular bone parts by means of the handle of a scalpel to facilitate the following decalcification of the superficial calcaneal trabecular layer, delineating from the surrounding trabeculae. Decalcification of the samples removed the hydroxyapatite leaving behind the collagen scaffold that connects the AT and the PF. This allowed to investigate the mechanical properties of the collagenous scaffold that connects the AT and the PF and compare it to the two structures. Following the hydroxyapatite removal, the collagen scaffold mechanically behaves in a soft tissue-like manner. For the decalcification of calcaneal samples, the resulting complexes of AT and PF connected by the most superficial inferior and posterior calcaneal trabeculae were submerged into a 10wt% ethylene-diamine-tetra-acetate (EDTA) solution for four weeks with a weekly renewal. Then, dog bone-shaped samples adapted from an ISO-527–2 template^13^ for biomechanical testing were cut from the AT-calcaneus-PF complex according to Fig. 1A. Samples for biomechanical testing were retrieved along the AT, calcaneus and PF along a medial and a lateral line based on the following morphological observations: the median fibrous septum of the soleus muscle (separating the AT fibre bundles into a medial and lateral line), median fibrous septae proximal to the most proximal insertion of the AT into the calcaneus (symmetrically separating the AT into medial and lateral bundles shown in histology sections) and finally the division of the PF into two larger bundles (central and lateral, the medial bundle was neglected here due to its smaller size). Along the medial and lateral lines, the following corresponding pairs were retrieved: MPAT (medial proximal AT) and LPAT (lateral proximal AT), MDAT (medial distal AT) and LDAT (lateral distal AT); MB (medial bone) and LB (lateral bone); MCP (medial calcaneal periosteum) and LCP (lateral calcaneal periosteum); MPPF (medial proximal PF) and LPPF (lateral proximal PF) and MIPF (medial intermediate PF) and LDPF (lateral distal PF). The MDPF (medial distal PF) of the medial line did not have a lateral line counterpart.

### Uniaxial quasi-static mechanical testing

The samples’ cross-sectional areas were determined by creating polysiloxane (medium-bodied, Exahiflex; GC Corporation, Tokyo, Japan) cross section casts, which were subsequently scanned (Perfection 7V750Pro; Seiko Epson Corporation, Suwa, Japan) and computed by means of the Measure 2.1d software (DatInf, Tübingen, Germany). Self-printed clamps with sharp pyramids to prevent specimen slippage during tensile testing were used (Fig. 1B)^14^. Before tensile testing was conducted, the samples were speckled using a black pencil to create a randomly distributed pattern, necessary for the digital image correlation (DIC) (Fig. 1C). A uniaxial testing machine (Allround Table Top Z020; Zwick Roell, Ulm, Germany) equipped with an Xforce P load cell (2.5 kN; Zwick Roell, Ulm, Germany) was used to conduct the tensile tests at room temperature. The testControl II software (Zwick Roell, Ulm, Germany) was used. Twenty load-unload preconditioning cycles with a force range of 0.5 to 2.0 N were applied before the tissues were stretched until failure (Fig. 1C–E). All tissues were strained in the longitudinal axis according to the samples’ predominant collagen orientation. The displacement rate was 20 mm/min and the sample reading rate was 100 Hz. A single-charge coupled camera with a resolution of 2.8 Megapixels (Q400; Limess, Krefeld, Germany) and the ISTRA 4D software (VRS 4.4.1.354; Dantec Dynamics, Ulm, Germany) were used for strain data evaluation of the mechanical tests.

### Data processing and statistical analysis

**Figure 2:**
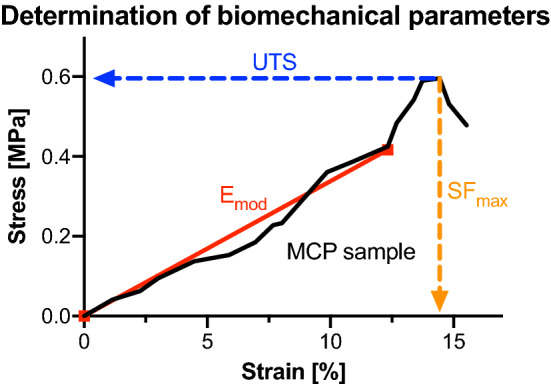
The determination of the biomechanical parameters in this study is shown representatively on a medial calcaneal periosteum (MCP) sample of this study. The elastic modulus (E_mod_) was determined by a linear regression analysis between the zero-point and the point that equals 70% of the maximum stress. The ultimate tensile strength (UTS) equals the maximum stress (F_max_) divided by the cross section value before the tissue failed when being stretched. The strain at maximum force (SF_max_) reflects how much the sample was strained at the point of the UTS compared to its initial length.

Synchronized force readings by MATLAB R2017b software (Mathworks, Natick, USA) and DIC data were used to calculate mechanical properties. Elastic modulus (E_mod_), strain at maximum force (SF_max_) and ultimate tensile strength (UTS) were evaluated (Fig. 2). For statistical evaluation, Excel version 16.15 (Microsoft Corporation, Redmond, USA) was chosen. Firstly, the biomechanical parameters of the subsamples were averaged according to their respective location (e.g., all MB values of the different tested samples were averaged) within the AT-calcaneus-PF complex. Following this, the averaged values of the subsamples were compared among the 13 different sites (e.g., MB vs. LB) and classified into the following groups according to being part of the percentile fractions shown in parentheses: low (0 to 24th percentile), below median (25th to 49th percentile), median (50th percentile), above median (51 to 74th percentile) and high (75th to 100th percentile).

### E12-sheet plastination and histology

The two E12-plastinated AT-calcaneus-PF complexes used in this study were taken from one entirely plastinated cadaver. The cadaver was embalmed according to Xu et al.^15^. The plastinates were scanned at 1200 dpi (Epson Perfection V750 Pro Scanner, Epson, Jakarta, Indonesia). For histological analyses, one fresh AT-calcaneus-PF complex was embedded into paraffin and horizontally sectioned with a section thickness of 20 µm using the same orientation as for the plastinates. Subsequently, the selected sections that corresponded to the plastinated slices were stained with hematoxylin eosin (H&E) and Masson–Goldner as trichromic staining (all consumables by Dr. Hollborn GmbH & Co KG, Leipzig, Germany) and photographed using a Zeiss Axioskop 40 (Carl Zeiss AG, Oberkochen, Germany) combined with an Olympus DP22 camera system (Olympus K.K., Shinjuku, Japan).

## Results

### Biomechanical mapping of the Achilles tendon-calcaneus-plantar fascia complex

**Figure 3:**
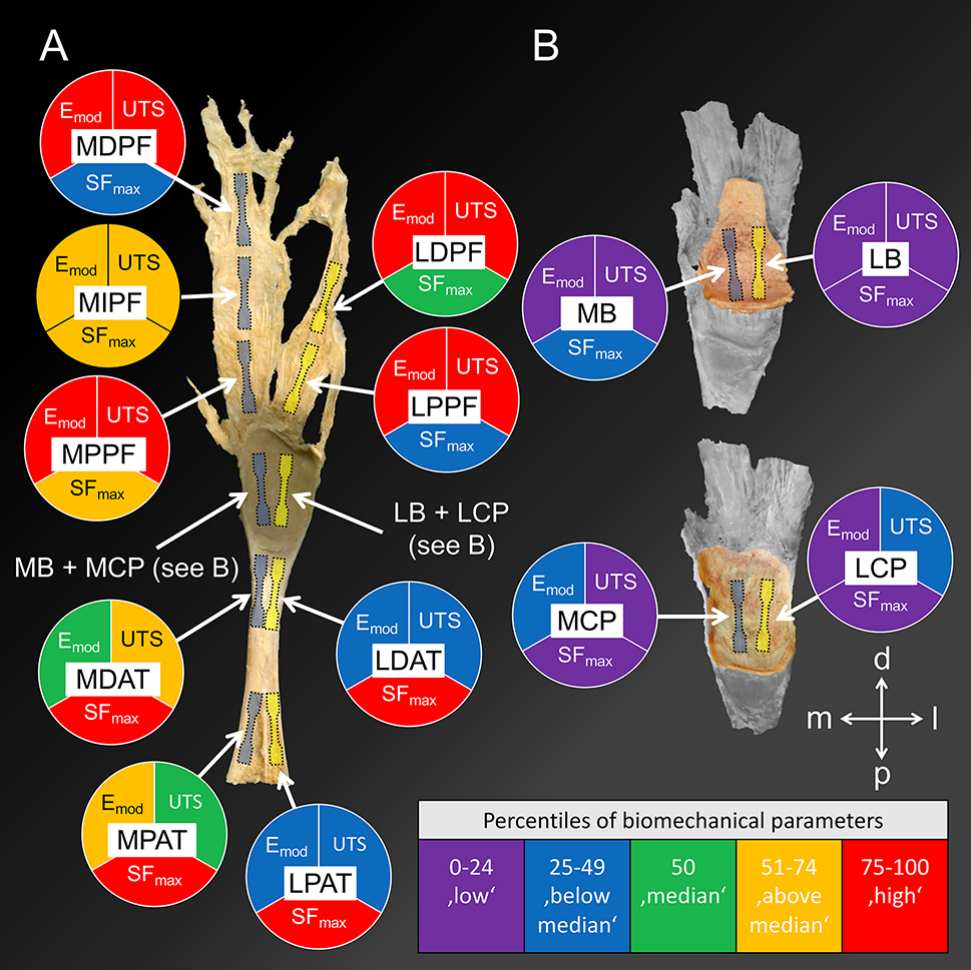
A graphical overview of the obtained biomechanical parameters and the retrieval site of the ‘dog bone’ samples from the Achilles tendon-calcaneus-plantar fascia complex is depicted. The colour code reflects the respective fraction of percentiles that the individual sample belongs to in comparison to all the other samples. (**A**) Samples of the decalcified human Achilles tendon-calcaneus-plantar fascia complex were retrieved according to a medial (blueish ‘dog bones’) and lateral (yellow ‘dog bones’) line, view from inside. MPAT, medial proximal Achilles tendon; MDAT, medial distal Achilles tendon; MPPF, medial proximal plantar fascia; MIPF, medial intermediate plantar fascia and MDPF, medial distal plantar fascia. The following samples were retrieved from the lateral line from proximal to distal: LPAT, lateral proximal Achilles tendon; LDAT, lateral distal Achilles tendon; LPPF, lateral proximal plantar fascia and LDPF, lateral distal plantar fascia. (**B**) A medial bone (MB) and medial calcaneal periosteum (MCP) sample were retrieved. Equally, a lateral bone (LB) and lateral calcaneal periosteum (LCP) sample were retrieved. d, distal; E_mod_, elastic modulus; l, lateral; m, medial; p, posterior; SF_max_, strain at maximum force; UTS, ultimate tensile strength.

All 76 tested subsamples withstood the tensile tests conducted in this study. Low elastic moduli were noted in the decalcified calcaneal samples (MB: 4 MPa; LB: 7 MPa) and the lateral calcaneal periosteum sample (LCP: 16 MPa). High elastic moduli were found exclusively in the PF (MPPF: 118 MPa; LDPF: 142 MPa; MDPF: 144 MPa) with the highest value observed in the lateral proximal PF (LPPF: 192 MPa). The UTS was low for the decalcified calcaneal samples (MB: 0.4 MPa; LB: 0.6 MPa) and the medial calcaneal periosteum sample (MCP: 2 MPa). As noted for the elastic moduli, high tensile strengths were exclusively found in the PF samples (MDPF: 22 MPa; LDPF: 24 MPa; LPPF: 29 MPa) with the highest value observed in the medial proximal PF (MPPF: 29 MPa). The SF_max_ was low in the calcaneal periosteum samples (MCP: 15%; LCP: 16%) as well as in the lateral decalcified bone sample (LB: 16%). High SF_max_ values were noted in all tested AT samples (MPAT: 31%; LPAT: 35%; MDAT: 34%; LDAT: 42%). The biomechanical parameters are graphically depicted in Fig. 3.

### Macroscopic observations of the Achilles tendon insertion into the calcaneus

**Figure 4:**
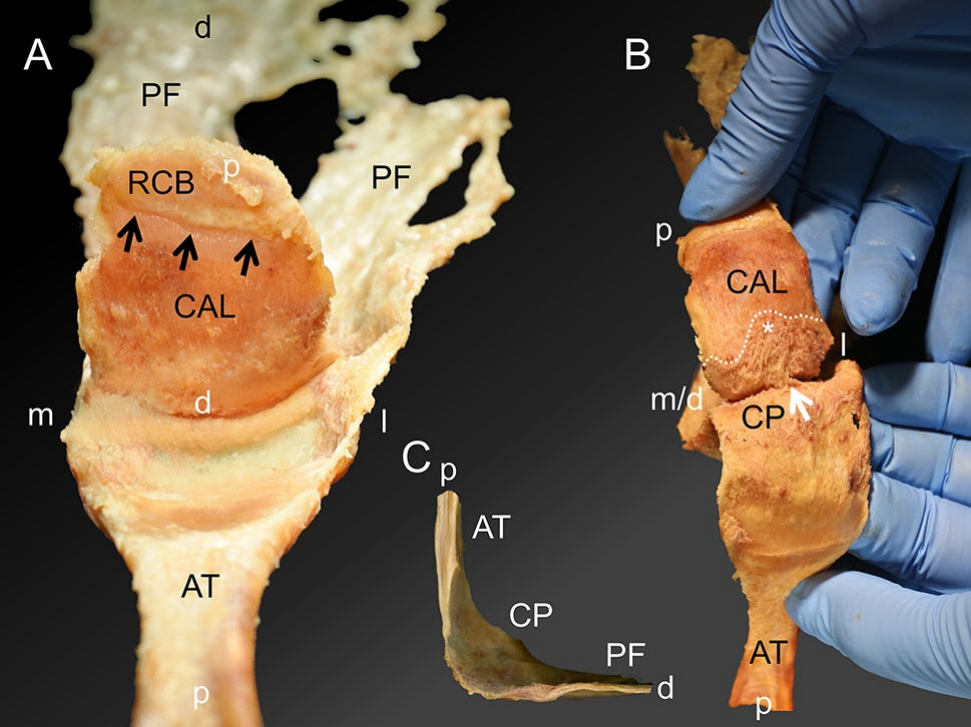
Macroscopic observations during the separation of the Achilles tendon-calcaneus-plantar fascia complex (AT-calcaneus-PF complex). (**A**) The AT-calcaneus-PF complex can be observed looking from proximal to distal after the AT was separated from its weak attachment on the calcaneus and reverted backwards. (**B**) The calcaneal tuberosity becomes visible if the separation of the AT from the calcaneus is continued. The white arrow points at the median fibrous septum originating from the calcaneus and contributing to the calcaneal periosteum, which is depicted more proximally in the plastinate and histology sections shown in Fig. 7. (**C**) The macroscopic transition of ligamentous structures between the AT and the PF is shown after the calcaneus was removed (view from medial to lateral onto a sagittal plane after the AT-calcaneus-PF complex had been longitudinally divided into two parts). AT, Achilles tendon; black arrows, transverse insertion line; CAL, calcaneus; CP, calcaneal periosteum; PF, plantar fascia; RCB: retrocalcaneal bursa, white asterisk, most proximal point of the calcaneal tuberosity; d, distal; m, medial; l, lateral; p, proximal.

The AT weakly attached to the upper posterior calcaneus between the proximal retrocalcaneal bursa and the distal calcaneal tuberosity (lower calcaneus) (Fig. 4A). After the AT was detached, a smooth surface remained on both the AT and calcaneus within the area of the former attachment (Fig. 4A). Continuing the separation of the two structures distally, the area of the calcaneal tuberosity revealed a stronger connection between the AT and the calcaneus, leaving a frayed surface on both the calcaneus and the opposite detached thin layer of calcaneal periosteum (Fig. 4B). The proximal border of the calcaneal tuberosity ran in a wavy fashion with the most proximal point in the mid of a mediolateral axis. After AT and PF are separated from the calcaneus the calcaneal periosteum connects the two structures in a bridge-like manner (Fig. 4C).

### Morphological observations of the Achilles tendon-calcaneus-plantar fascia complex based on plastinated slices

**Figure 5:**
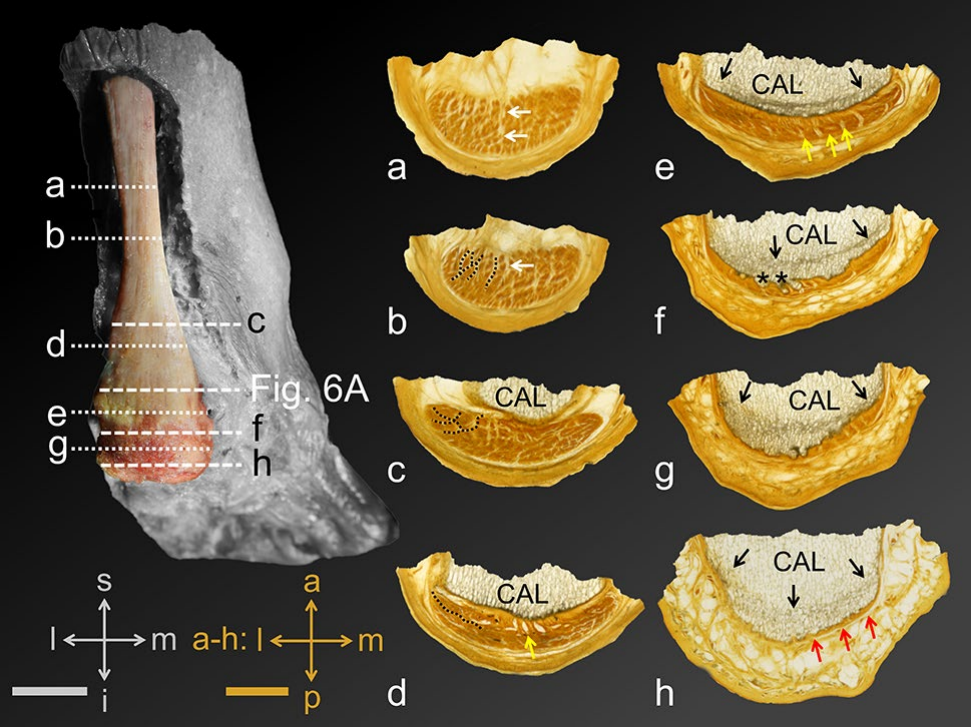
Plastinated sections of the Achilles tendon (AT) and its insertion into the calcaneus are depicted in the transverse plane. Section levels (a-h) are illustrated on a dissected AT shown from dorsal on the left. (**a**) Section level: upper posterior subtalar joint, (**b**) Section level: posterior subtalar joint, (**c**) Section level: uppermost part of the posterior calcaneal surface, (**d**) Section level: transition between the upper and middle part of the posterior calcaneal surface, (**e**) Section level: middle part of the posterior calcaneal surface, (**f**) Section level: lower part of the posterior calcaneal surface (calcaneal tuberosity), (**g**) Section level: lower posterior calcaneal surface, (**h**) Section level: proximal end of the heel fad pad; Black arrows, trabecular bone layer appearing to be denser compared to the surrounding tissue; black dotted lines, thin fibrous AT septae; CAL, calcaneus; red arrows, thin AT layer covering the calcaneus; white arrows, median septum of soleus muscle; black asterisks, enthesophytes; yellow arrow, thick fibrous septae; a, anterior; i, inferior; l, lateral; m, medial; p, posterior; s, superior; Scale bars: grey, 24 mm; orange, 6 mm.

**Figure 6:**
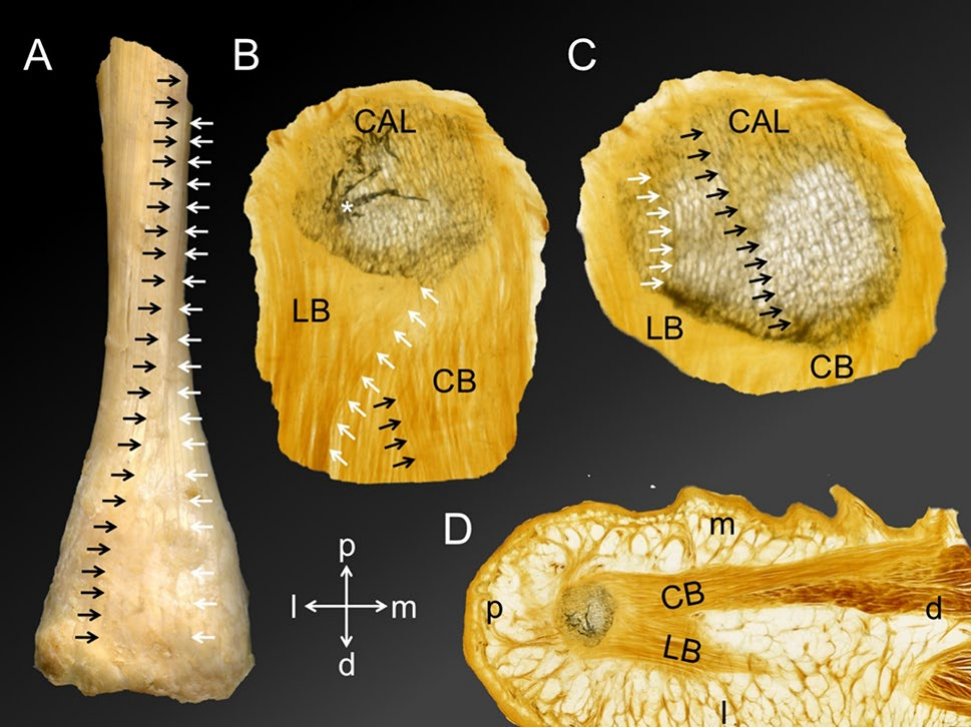
The course of both Achilles tendon (AT) and plantar fascia (PF) collagen fascicles is depicted**. **(**A**) A dissected AT is shown from dorsally. The black arrows indicate a collagen bundle running from proximal medial to distal lateral in the superficial AT layer. In contrast, the white arrows indicate that the most medial superficial fascicles of the proximal AT stay medial in the distal part of the AT. (**B**) A plantar view on the most inferior tip of the calcaneus (CAL) is displayed with the inserting lateral (LB) and central bundle (CB) of the PF. The white arrows point on collagen fibres that originate from the medial CAL taking a course towards the LB. Inversely, a number of fibres can be observed taking a medially directed course (indicated by the black arrows). The white asterisk indicates a bony artefact. (**C**) A plantar view on the CAL is displayed at a slightly more proximal level compared to B. Focusing on the trabecular architecture, the black arrows indicate representative trabeculae that take a lateromedial course, similar to the more distal fibres of the CB shown in B. Similarly, the white arrows highlight representative trabeculae that are in line with the orientation of the LB. (**D**) An overview of the slice shown in B is depicted, showing the CB and LB originating from the CAL taking a distal course (view from plantar). Directions A-C: indicated by a cross; d, distal; l, lateral; m, medial; p, proximal.

In the proximal AT, the median septum of the soleus muscle was identified separating the AT into medial and lateral fibres (Fig. 5a, b). The AT-calcaneus-PF complex revealed a course change of the fibrous septae within the AT from running perpendicular to the anterior AT border proximally (Fig. 5b), running oblique to it more distally (Fig. 5c), and parallel to the anterior AT border directly proximal of the proximal AT insertion into the calcaneus (Fig. 5d). At the latter level, fibrous septae were depicted at the midpoint of a mediolateral AT axis. Fibrous septae within the AT seemed to continue in the superficial calcaneus within a superficial trabecular area that differed from the rest of the calcaneal trabeculae in terms of a darker appearance (Fig. 5e). These darker trabeculae changed from being a superficial band at the level of the proximal AT insertion (Fig. 5e) to being a demarcating line only with regular calcaneal trabeculae between the aforementioned and the calcaneal surface distally (Fig. 5f–h). At the level of the calcaneal tuberosity, several finger-shaped bony extensions were observed, yielding a regular trabecular architecture (Fig. 5f), compatible with the definition of enthesophytes. These areas seemed to develop in the areas of the fibrous AT septae (Fig. 5f). The inspection of the superficial AT indicated a distinct crossing of collagen fibres from proximal medial to distal lateral (Fig. 6A). In the more distal medial AT, straight superficial fibres without a crossing of sides were observed (Fig. 6A). Deeper calcaneal layers revealed a trabecular course, which is in line with the orientation of the more superficially observed collagen orientation of the medial and lateral bundle of the PF (Fig. 6B–D).

### Morphological observations based on histology

**Figure 7:**
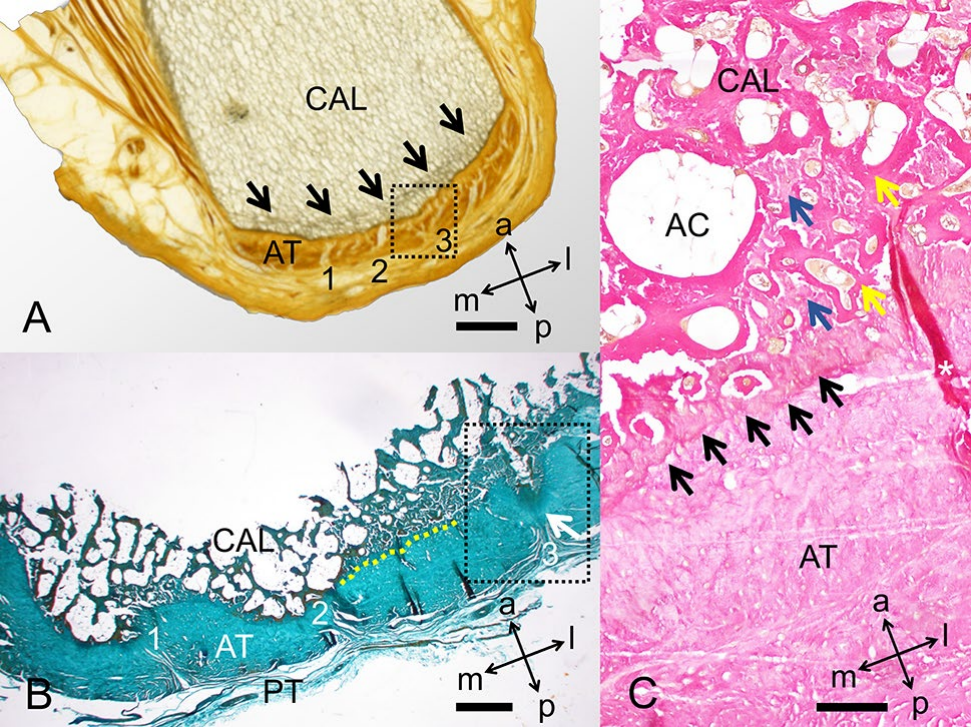
The insertion of the Achilles tendon (AT) into the calcaneus (CAL) is depicted on plastinates and in histology. (**A**) Plastinated slice. Black arrows: demarcated trabeculae; dotted box: corresponds to an area similar to the dotted box in B; 1–3: fibrous septae, similar to the ones in B. Scale bar: 4 mm, (**B**) Masson–Goldner stain. Black box, median fibrous septum; PT, paratenon; 1–3: fibrous septae, similar to the ones in A; white arrow: median calcaneal prominence; yellow dotted line: demarcation between AT and CAL. Scale bar: 1 mm, (**C**) H&E stain. AC, adipocytes (located in bone marrow); black arrows: border between AT and CAL in greater detail compared to B; blue arrows: collagen bundles; white asterisk: folding artefact; yellow arrows: calcaneal trabeculae. Scale bar: 200 μm; a, anterior; l, lateral; m, medial; p, posterior.

Fibrous septae seen in plastinates (Fig. 7A, corresponding to the level of the lower part of the posterior calcaneal surface in Fig. 4 and illustrated as orientation line in Fig. 5) are running between the calcaneus and the AT paratenon perpendicularly to the course of the remaining AT fibre bundles as indicated in the Masson–Goldner stain in greater detail (Fig. 7B). The median fibrous septum originating from a slight median calcaneal prominence symmetrically separates the most medial running longitudinal AT fibre bundles (Fig. 7B, yellow dotted line). Based on H&E slices, the demarcating superficial trabecular layer within the calcaneus seen on plastinates consists of calcaneal trabeculae surrounded by collagens and adipocytes (Fig. 7C).

## Discussion

Successful stretching exercises involving the AT when treating various forms of heel pain including plantar fasciitis have highlighted the functional connection between AT and PF^2^. Beyond that, highly regular interindividual trabecular arrangements in the superficial posterior and inferior calcaneus, which seem to form an osseous bridge between AT and PF suggested that this connection might exist even morphologically^7^. The given study provides evidence in favour of a morphological entity of the AT, the superficial posterior and inferior calcaneus and PF based on plastination and histology. The sheet plastination sections assessed in this study revealed that the diminution of the AT when inserting into the calcaneus from proximal to distal is associated with the occurrence of a delimitable highly regular trabecular band in the superficial posterior and inferior calcaneus, which differs in colour intensity from the surrounding hard tissue and seems to continue the course of the AT towards the PF. The histological observations revealed that this band consists of calcaneal trabeculae, which are surrounded by collagens and adipocytes. This is compatible with the concept of a formerly continuous tendon, that ossified in the area of the calcaneus resulting in two separated tendons, namely the AT and PF. Fibrous septae within the AT seemed to continue their course in the aforementioned area of regular trabecular arrangement. In more distal plastinated slices, a trabecular orientation within the calcaneus was noted, which matched the orientation of the adjacent PF fibres. It is conceivable that predominantly during movements the mechanical stimuli from the AT proximally and the PF distally contribute to the ossification of the tendon into the bone at the interface between the AT/PF and the calcaneus. The observed enthesophytes might be an example that in certain cases this metaplasia might happen to a greater extent than ‘normally’, again being closely connected to the structural organization of the tendons (in the given study the AT). Supporting this, the prevalence of calcaneal spurs was shown to increase with age with no sex-dependency^16,17^. Reciprocally, the observed differences in colour intensity within the superficial calcaneal regular trabeculae itself might be an indication that the load distribution is irregular when mechanically strained. Consequently, the relation of trabeculae, tendinous collagens and fat might vary in different regions of these highly regular trabeculae ranging from a dense dark greyish line to an ‘inconspicuous’ trabecular architecture in other plastinated sections as observed in this study.

This is the first study to map the load-deformation characteristics of the entire AT-calcaneus-PF complex using a uniaxial tensile testing setup. Decalcification of the calcaneal samples for the mechanical tests was conducted, to remove all hydroxyapatite components, leaving behind a collagen scaffold, which allowed to use an identical tensile testing protocol for hard and soft tissues (Scholze, et al. 2018). Thereby, it is possible to focus on the bone component that might have been part of the continuous AT-PF tendon before it ossified in the area of the calcaneus. The pooled UTS of all samples, describing the applicable maximum stress a material can withstand before breaking when stretched, showed that the decalcified bone samples were at the lower end of the obtained values. This can be interpreted from different perspectives. If it is assumed that the highly regular trabeculae of the AT represent a formerly continuous tendon, which subsequently ossified, the organic matrix might have dismounted a substantial amount of the longitudinally oriented collagens while bricking the same. Consequently, this resulted in a low tensile strength when tested in the given setup. The group of Snow et al. stated that in neonates thick continuous fibres were observed between AT and PF, which decreased during life to ultimately, apart from the calcaneal periosteum, completely disappear in late adulthood^7^. However, the given study revealed oppositional results as the decalcified calcaneal samples originating from an old adulthood group with a mean age of 74 years without a single failing specimen withstood the uniaxial tensile tests conducted in this study. This evidences a certain amount of structural integrity of the collagenous scaffold, which is independent of the calcaneal ossification. Overall, the morphological results of the given study favour an initial continuous tendon between AT and PF that becomes integrated into the superficial posterior and inferior calcaneus during life. In the given study the ‘bridging’ was detectable histologically in a sample retrieved from a cadaver at the end of the second decade of life indicating that the subsequent ossification at the calcaneal level should have been finished in the younger adulthood already. There seems to be a continuous ossification of the AT as a proximal movement of the AT insertion with age was shown based on MRI datasets^18^. Another possibility is that the high UTS of the tendinous structures observed in the given study compared to the decalcified bone samples is the result of a collagen increase in the AT and PF in relation to the collagenous structure that becomes integrated into the calcaneus early in life. This could be caused by the repetitive mechanical stimuli generated during walking or jumping, consequently strengthening both AT and PF^19,20^. On the contrary, based on the observed low UTS values of the decalcified bone samples in this study it could be argued that a tendinous connection between the AT and PF, being integrated into the calcaneus later in life, was never existing and the observed longitudinally trabecular structure that seems to connect the AT and the PF only represents a structural adaptation of the calcaneal bone to the pulling forces of the AT and the PF into oppositional directions. The E_mod_ of the decalcified calcaneal samples quantitatively reached roughly a quarter of the values stated for comparable soft tissue scaffolds in an identical testing setup^21,22^. The higher elasticity (meaning lower elastic moduli) of the decalcified bone samples compared to all other soft tissues in this study resembles the free spaces that occurred after the removal of the organic matrix, allowing the remaining collagen scaffold to expand into the same. In contrast, AT and PF samples revealed higher values, most likely caused by their dense collagenous organization resulting in both higher elastic moduli and tensile strengths.

Several authors indicated a twist of the AT-forming fascicles on their course from the origin at the medial/lateral gastrocnemius and soleus muscle to the insertion at the calcaneus^23–27^. The macroscopic observations in the given study support the concept of a twisting AT based on the changing arrangement of AT septae from proximal to distal noted in plastinated sections and the mediolateral course of superficial posterior AT fibres seen in the dissected samples. Also, an AT fibre twist might explain that the most proximal point of the calcaneal tuberosity ended up at the midpoint of a mediolateral calcaneal axis in this study, similar to the bulking middle part when twisting a sheet of paper. According to Szaro et al.^26^ the medial gastrocnemius fibres are located posteriorly (superficially), the lateral gastrocnemius fibres anteriorly (deeply) and the soleus muscle fibres medially/centrally in the AT. None of the aforementioned groups followed the course of AT fibres beyond the calcaneal insertion as it was done here for the first time to our knowledge. This study indicated the feasibility to subdivide the AT-calcaneus-PF complex into numerous subsamples, allowing to use a uniform uniaxial quasi-static testing protocol for both soft and hard tissues at the same time. Due to the limited amount of available tissues for the given study the authors refrained from performing correlation analyses, which in a larger sample size might reveal information about the structural organization of the AT-calcaneus-PF complex from a material testing point of view. Thus, applying the given setup investigating the AT-calcaneus-PF complex using a medial and lateral line could shed light on the complex reorganization of collagen bundles from proximal (calf) to distal (sole). Also, the possible impact of the collagen bundle rearrangement on the mechanical properties of the AT-calcaneus-PF complex in a larger sample size might enable to draw conclusions on the structural organization of this complex. Further investigations with regards to age, sex and bodyweight deploying a larger sample size might provide further insights into the here stated site-dependent biomechanical mapping of the human AT-calcaneus-PF complex. Using larger sample sizes the here stated site-dependent biomechanical mapping of the human AT-calcaneus-PF complex can be applied to investigate the biomechanical properties across different species. This might provide further insights into the evolutionary anatomy of these structures^8^. This given study provides evidence for a structural continuity of the AT and the PF via the posterior calcaneal trabeculae. It should be noted that the plantaris longus muscle has been shown to be de-coupled from the PF in humans and, therefore, lost its potential to strengthen the former^8^. This has been achieved by a firm attachment of the plantaris muscle tendon to the calcaneal periosteum^8^. The former concept of immobilizing the spatially closely related plantaris muscle tendon appears to be similar for the AT-calcaneus-PF complex discussed in this given study.

## Limitations

A number of limitations have to be addressed for this study. Firstly, the minute samples size was limited by the number of available tissues. The mechanically tested samples in this study were embalmed using a phenoxyethanol-based fixative^12^ and subsequently decalcified by means of a 10wt% EDTA solution, which might have influenced the biomechanical properties. However, the biomechanical purpose of the given study was to compare the biomechanical properties of several sub-structures of the AT-calcaneus-PF complex to each other with special interest of the change of the individual parameters from proximal to distal rather than giving a detailed lifelike material description of the aforementioned. The bias introduced by the embalming and decalcification likely influenced all samples to a comparable amount as a systemic error and should, therefore, not influence the relative relationship between the biomechanical properties of the tested subsamples. The study intended to test the calcaneal samples within the area of the regular trabecular arrangement, which possibly reflects the former tendinous part. In this regard, for the preparation of calcaneal samples for mechanical testing, the cancellous bone outside the area of the highly regular trabecular arrangement might not have been removed entirely in all samples, which potentially influenced the acquired material parameters. Furthermore, the elastic modulus was here evaluated in the linear part of each nominal stress–strain curve, using a regression analysis. Human soft tissues, however, naturally present a non-linearity concerning their load-deformation behaviour. Therefore, the presented values are suitable for the given purpose but likely represent an oversimplification when these parameters are applied for, e.g., lifelike computer simulations of human soft tissues. The post mortem interval of the tissues in this study is unknown. However, no obvious signs of tissue degradation such as skin discolouration were noted, which would indicate a prolonged post mortem interval. It remains unknown at this stage whether the EDTA affected the biomechanical properties of the extracellular matrix components of the scaffold, resulting from hydroxyapatite removal, e.g., by destruction of glycosaminoglycans or collagens.

## Conclusions

Morphological observations support the concept of a collagen fascicle continuity between the AT and the PF via the superficial posterior and inferior calcaneus according to a functional and morphological ‘AT-calcaneus-PF complex’. A biomechanical testing setup was established that allows to uniformly test the involved hard and soft tissues of the AT-calcaneus-PF complex in a larger sample size to reveal structural information of this complex from a material testing point of view in the future.
